# Intervention in the Native and Transplant Liver in Children: An Update

**DOI:** 10.1007/s00270-025-04288-0

**Published:** 2025-12-07

**Authors:** Stephanie Franchi-Abella, Paolo Marra, Eric J. Monroe, S. Murthy Chennapragada

**Affiliations:** 1 Department of Pediatric Radiology, DMU Smart Imaging, Bicêtre Hospital, AP-HP, Le Kremlin-Bicêtre, France; 2 Reference Centre for Vascular Diseases of the Liver, FSMR FILFOIE, ERN RARE LIVER, FHU Hépatinov, Le Kremlin-Bicêtre, France; 3 BIOMAPS UMR 9011 CNRS – INSERM – CEA, Paris-Saclay University, Le Kremlin-Bicêtre, France; 4 School of Medicine and Surgery, University of Milano-Bicocca, Piazza dell’Ateneo Nuovo 1, 20126 Milan, Italy; 5 Department of Radiology, ASST Papa Giovanni XXIII, Bergamo, Italy; 6 Vascular and Interventional Radiology, Department of Radiology, University of Wisconsin, 600 Highland Ave, Madison, WI 53792 USA; 7 Department of Paediatric Interventional Radiology, The Children’s Hospital at Westmead, Sydney Children’s Hospital Network, Westmead, NSW Australia; 8 Sydney Medical School, University of Sydney, Camperdown, NSW Australia

**Keywords:** Angioplasty, Cholangiography, Congenital portosystemic shunt, Portal hypertension, Transjugular intrahepatic portosystemic shunt, Stents

## Abstract

Interventional radiology (IR) is rapidly expanding in paediatric care due to technological advancements and research, as well as its minimal invasiveness compared to surgery. New therapeutic options have been developed to manage both benign and malignant conditions refractory to medical treatments or for which surgery has failed or is not feasible, particularly regarding locally advanced liver tumours, vascular malformations and vascular tumours of the liver, lymphatic disorders, portal hypertension and complications of liver transplantation. A multidisciplinary approach is essential for the optimal management of patients, as well as for increasing the awareness of paediatricians and surgeons regarding IR. This review article will discuss the pathologies affecting both the native and the transplant liver with focus on cutting-edge devices that have significantly influenced clinical practice, gaps of knowledge, research needs and potential collaborations.

## Introduction

The article systematically describes the main benign and malignant conditions affecting the native or transplanted liver that have an interventional radiology treatment option. Each paragraph is dedicated to a specific condition and its corresponding treatment, with an analysis of the current scientific evidence and the most innovative techniques. A final paragraph outlines the limitations of current knowledge, the areas where paediatric-specific devices are needed, and fields for further research.

### Hepatoblastoma

Transarterial chemoembolization (TACE), transarterial radioembolization (TARE), and percutaneous ablative therapies—such as high-intensity focused ultrasound (HIFU), microwave ablation (MWA), cryoablation, and radiofrequency ablation (RFA)—are well established in adult liver oncology but remain rarely applied to paediatric hepatoblastoma and other malignant liver tumours, mainly serving as bridge to liver transplantation or surgery.

An growing body of literature is paving the way for a new era, in which IO may play a pivotal role for the primary management of liver malignancies in children. Much of the knowledge, especially regarding technical aspects, can be extrapolated from clinical experience in adults; however, paediatric patients deserve specific attention and periprocedural care.

Current literature describes 87 cases of paediatric hepatoblastoma treated with TACE alone [1–10], mostly including patients under age 5. Additionally, 41 cases were treated with TACE combined with percutaneous ablative therapies [11–13] in patients under 5, and 42 cases with RFA in patients under 17 [14, 15]. Five chemoresistant patients under 5 underwent TARE with Yttrium-90, followed by surgery [16, 17]. The drug-eluting bead technique, common in adults, has been reported in only one case report regarding a 13-month-old infant [9].

A meta-analysis by Zambaiti [18], of patients aged 0–18, found that interventional radiology (IR) significantly reduced mortality rates (43 vs. 71%). TACE effectively reduces tumour size, AFP levels, and operative time, thereby improving resection rates, prognosis, and 2-year tumour-free survival [1, 3, 7, 18].

Disease control was achieved in PRETEXT III and IV hepatoblastomas treated with TACE + HIFU and chemotherapy, particularly in 10/12 patients under age 5 in Wang’s series and 4/8 patients under 4 in Chen’s cohort [12, 13]. However, increased mortality was observed in patients who did not undergo surgery, with 4 of 6 children whose parents refused surgery ultimately succumbing to the disease. Disease control was also achieved in patients without metastases treated with local therapy [1–4, 7]. TARE with Yttrium-90 facilitated local control in chemoresistant tumours [16, 17].

Complications associated with transarterial treatments mainly include postembolization syndrome (pain, nausea, fever, transient hepatic dysfunction). Minor rib deformations have been reported with HIFU [13]. The level of evidence of all the studies is limited by their retrospective nature and lack of control groups in most cases.

### Hypervascular Liver Tumours

Besides transarterial embolization (TAE) for haemostasis in the context of hepatic tumour rupture—most commonly hepatoblastoma—arterial interventions on hypervascular liver tumours are primarily used for arterial deprivation of life-threatening hepatic haemangiomas in infants and for the treatment of focal nodular hyperplasia (FNH).

Hepatic haemangiomas, either congenital or infantile, are the most common benign liver tumours in infants. They are typically asymptomatic and may regress spontaneously. However, in some cases they may lead to life-threatening complications such as high-output cardiac failure, consumptive coagulopathy, compartment syndrome, or significant arteriovenous shunts [19–21]. When they are symptomatic, medical management is the first-line treatment and when it fails IR (or surgery) are undertaken in a limited group of patients. Factors associated with the need for, and potential failure of medical therapy include the type of haemangioma, diffuse haemangiomas, large focal hemangiomas (> 40 mL), systolic velocity of the feeding artery > 110 m/s, hepatic vein dilatation, and the presence of porto-hepatic or arterio-portal shunts [19, 20]. If medical treatment is unsuccessful, suppression of the feeding arteries is required. Surgery was the initial approach until the early 1980s, when the first reported cases of TAE demonstrated its efficacy [22].

The goal of TAE is to selectively obliterate most of the arteries supplying the haemangioma(s) to decrease cardiac output and improve cardiac function. While the hepatic arteries are usually involved, extrahepatic arteries can also make significant contributions. In neonates, the umbilical arteries and veins can be used as vascular access points, preserving the femoral or axillary arteries and thereby reducing the risk of thrombosis [23–25].

Various embolic agents have been used for the treatment of hepatic haemangiomas, including polyvinyl alcohol (PVA) particles, coils, microparticles, plugs, *N*-butyl cyanoacrylate (NBCA), and ethylene vinyl alcohol copolymer (EVOH), with no consensus regarding the optimal choice [26–31]. In exceptional cases, transarterial chemoembolization (TACE) has been reported [32]. Given the variable angioarchitecture of these lesions, which may range from a simple arterial blush to complex cases with arterio-portal and/or porto-hepatic shunts, embolic agent selection should consider the potential risk of migration [33, 34].

The role of transarterial embolization (TAE) in the treatment of focal nodular hyperplasia (FNH) has evolved over time. Initially explored in the 1980s, interest in TAE declined until the past decade, when an increasing number of publications in adult patients reported encouraging results. However, no consensus has been reached regarding the most appropriate embolic agents [35–37]. Wang et al. [37] reported a large series of adult patients and found that the combination of lipiodol-based embolization (LBE) with polyvinyl alcohol (PVA) particles resulted in a significantly higher rate of complete remission compared to PVA particles alone.

In paediatric patients, only a few series and case reports are available [37–40], of which some involve both adults and children, with the majority of paediatric cases typically being post-adolescents. The main indication for TAE in children is large, symptomatic FNH deemed unresectable. Various embolic agents have been used, including polyvinyl alcohol, microspheres, and bleomycin, but no consensus exists regarding the most appropriate choice. In 2023, Yan et al. [38] published the largest paediatric series to date, comprising 17 patients treated for large (≥ 7 cm), symptomatic, unresectable FNH. Their protocol involved injecting LBE for distal and intralesional vascular embolization, followed by *N*-butyl cyanoacrylate (NBCA) for proximal arterial embolization.

Initial imaging identified factors predictive of the need for multiple TAE sessions, including lesions measuring ≥ 10 cm, proximity to the hepatic capsule, or location in the bare area of the liver. Vascularization by multiple collateral arteries—particularly extrahepatic arteries such as the right inferior phrenic artery, right internal thoracic artery, right renal capsular artery, and left gastric artery—was also associated with a higher likelihood of requiring multiple procedures if all arteries were not treated. No significant complications were reported. All 17 patients became asymptomatic, with a mean volume reduction rate of 96.9% (range 86–100%) and no recurrence at the last follow-up.

### Congenital Portosystemic Shunts (CPSS)

Nowadays IR plays a central role in the management of CPSS, although these malformations have been historically managed by surgery. IR is involved in sampling liver nodules and non-nodular parenchyma for histologic assessment, performing the occlusion test and occluding the shunt if technically feasible. There is increasing evidence that a hand-to-toe evaluation should be performed in patients diagnosed with CPSS, as even asymptomatic shunts may cause subclinical manifestations, mainly related to endocrine and neurocognitive impairment. In particular, closure of extrahepatic shunt is recommended also in asymptomatic patients [41].

For nodule characterization, image-guided needle biopsy should focus on various areas of the same lesion and on each nodule if multiple are present. This strategy enables a comprehensive histologic and molecular assessment of high-risk features that may indicate the need for surgical resection [41].

**Fig. 1:**
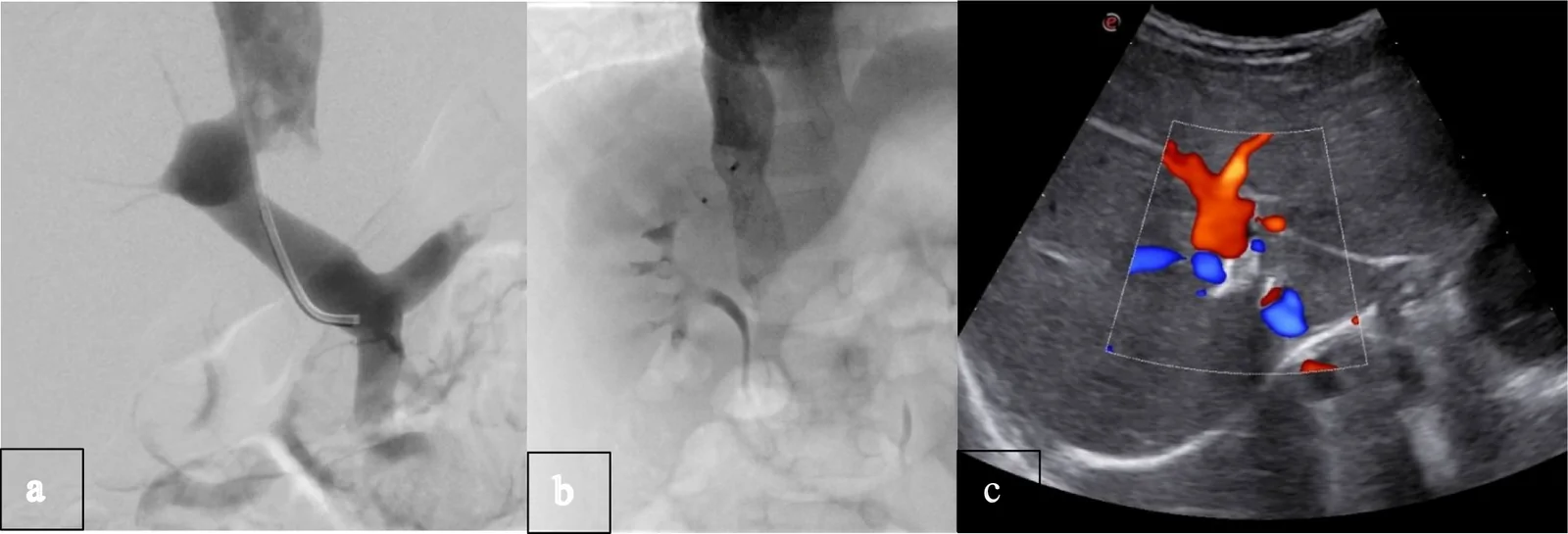
Endovascular congenital portosystemic shunt closure with a muscular ventricular septal occluder device. **a** Right transjugular anteroposterior digital subtraction venography image shows the short and wide porto-caval extrahepatic shunt with tiny intrahepatic portal branches. **b** Anteroposterior fluoroscopic venography after deployment of a muscular septal occluder device shows the correct placement of the plug without obstruction of the inferior vena cava. **c** Axial ultrasound image acquired 2 months after endovascular shunt closure shows regular colour signals within developed intrahepatic portal branches and patency of the inferior vena cava

Precise understanding of shunt anatomy is necessary to properly perform the occlusion test, aimed at assessing the suitability for shunt closure in one or two steps. If portal hypertension develops during the occlusion test, with absolute pressure rising above 30 mmHg or a portosystemic gradient above 20 mmHg, a staged closure is recommended to prevent serious post-closure complications [41]. Preoperative imaging is performed either by colour-Doppler and contrast-enhanced ultrasound in small children and infants without liver nodules, or with CT/MR angiography and MRI with hepatocyte specific agents for more complex anatomies or when characterization of liver nodules is required. For one-step closure, IR has a wide range of available devices ranging from coils, conventional plugs, with or without PTFE membranes within the nitinol mesh, to cardiologic devices, including septal and muscular defect occluders (Fig. 1). The right device must be chosen on a case-by-case basis, following assessment of the shunt morphology: while conventional Amplatzer plugs are suitable for long and narrow shunts or ductus venosus, septal occluders are better suited for short and wide shunts. Covered stents, intended to be deployed in the inferior vena cava to cover the shunt landing, may be also considered, although not recommended in children with a potential to grow [41].

Regarding staged closure, IR has limited tools as no devices are specifically labelled for partial closure of target vessels. If surgical expertise is not available in the centre, different techniques for standard device customization have been reported, as off-label use [42–44].

### Arterio-Portal Fistulas and Malformations

Intrahepatic arteriovenous fistulas are rare and are most often secondary to liver disease, trauma, or iatrogenic causes. When congenital, they are extremely rare, with fewer than 50 cases reported in the literature, and they primarily present as arterio-portal fistulas or, in more complex cases the term arteriovenous malformations is usually used. Congenital arteriovenous hepatic fistulas or malformations may be associated with predisposing factors such as Down's syndrome and hereditary haemorrhagic telangiectasia (HHT) [45].

Some forms may remain asymptomatic; however, these conditions typically manifest early in life, most often before the age of two, with signs of portal hypertension. The severity of portal hypertension is directly related to the degree of arteriovenous shunting. It can be severe, leading to ascites, gastrointestinal bleeding, diarrhoea, and failure to thrive in cases of significant arterio-portal shunting. The presence of portal hypertension is an indication for treatment. Several classification systems have been proposed based on the location or angiographic pattern of the malformation [46, 47]. In practice, as reported by Chaundry [48], simple arterio-portal fistulas with direct fistulous connections between an artery and the portal vein are more favourable for successful treatment. Conversely, complex arterio-portal fistulas with multiple intrahepatic and extrahepatic arterial feeders are more challenging to manage. Norton suggested that the presence of a complex form with an early clinical onset is a significant negative prognostic factor.

Treatment options include transarterial embolization (TAE) of the feeding arteries and embolization of the aneurysmal component of the portal vein, either through arterial access or percutaneously [46–51]. There is no consensus on the choice of embolization agents, with various materials reported in the literature, including coils, plugs, acrylic or polyvinyl alcohol microspheres, NBCA, ethylene vinyl alcohol copolymer (EVOH), and even a collagen–thrombin mixture [49–54]. The angiographic pattern should guide treatment decisions, with particular attention to preventing nontarget embolization, especially when using liquid agents, microparticles, or gelfoam. Some authors recommend anticoagulation, as occlusion of the arterio-portal shunts may initially lead to stagnation in the portal vein and subsequent thrombosis [48].

In cases where radiological treatment fails, surgery may be necessary to control the disease. For diffuse disease, liver transplantation may be the only curative option.

### Transjugular Intrahepatic Portosystemic Shunt (TIPS) and Portal Vein Recanalization (PVR) for the Management of Portal Hypertension

**Fig. 2:**
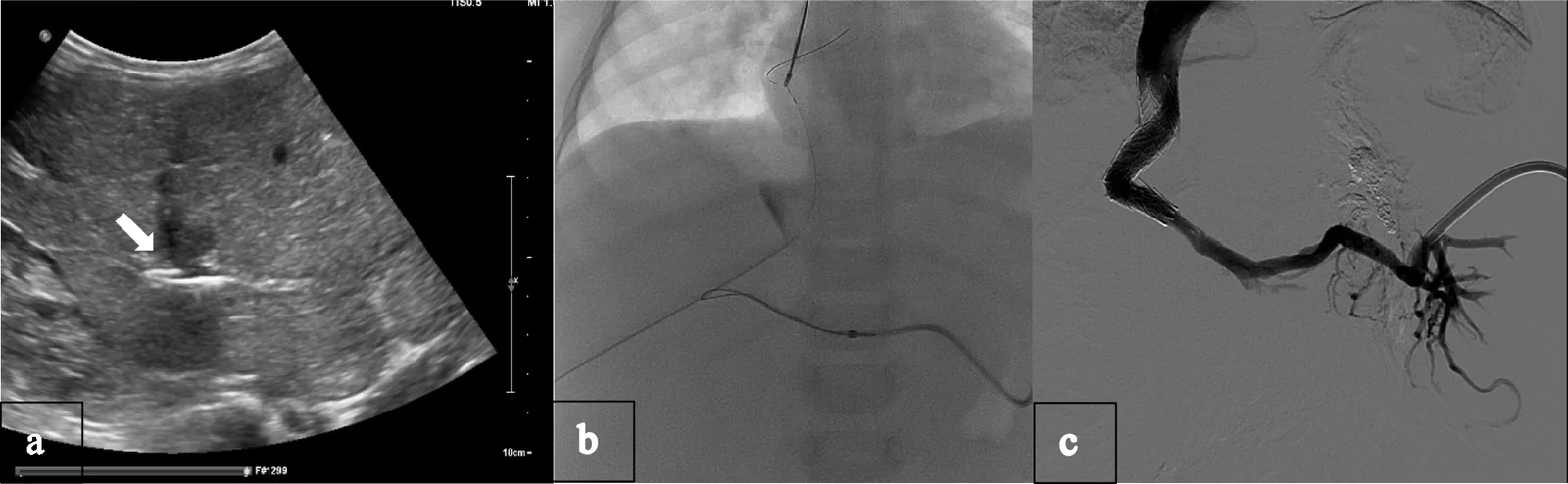
TIPS placement by combined transhepatic–transplenic–transjugular approaches using the “gun-sight” technique. A 2-year-old girl with portal hypertension resulting from congenital hepatic fibrosis. **a** Axial ultrasound image shows the transplenic snare in the right portal vein (arrow). **b** Anteroposterior fluoroscopic image shows the transhepatic targeting with a 22-gauge needle of the snare with coaxial puncture of the inferior vena cava to advance a transhepatic–transjugular wire; the distal end of the wire will be pulled from the splenic access to achieve a though-and-through transjugular–transplenic wire to accomplish the procedure from the transjugular route. **c** Anteroposterior digital subtraction venography image acquired after TIPS placement and glue embolization of gastroesophageal varices shows effective decompression of the portal circulation

**Fig. 3:**
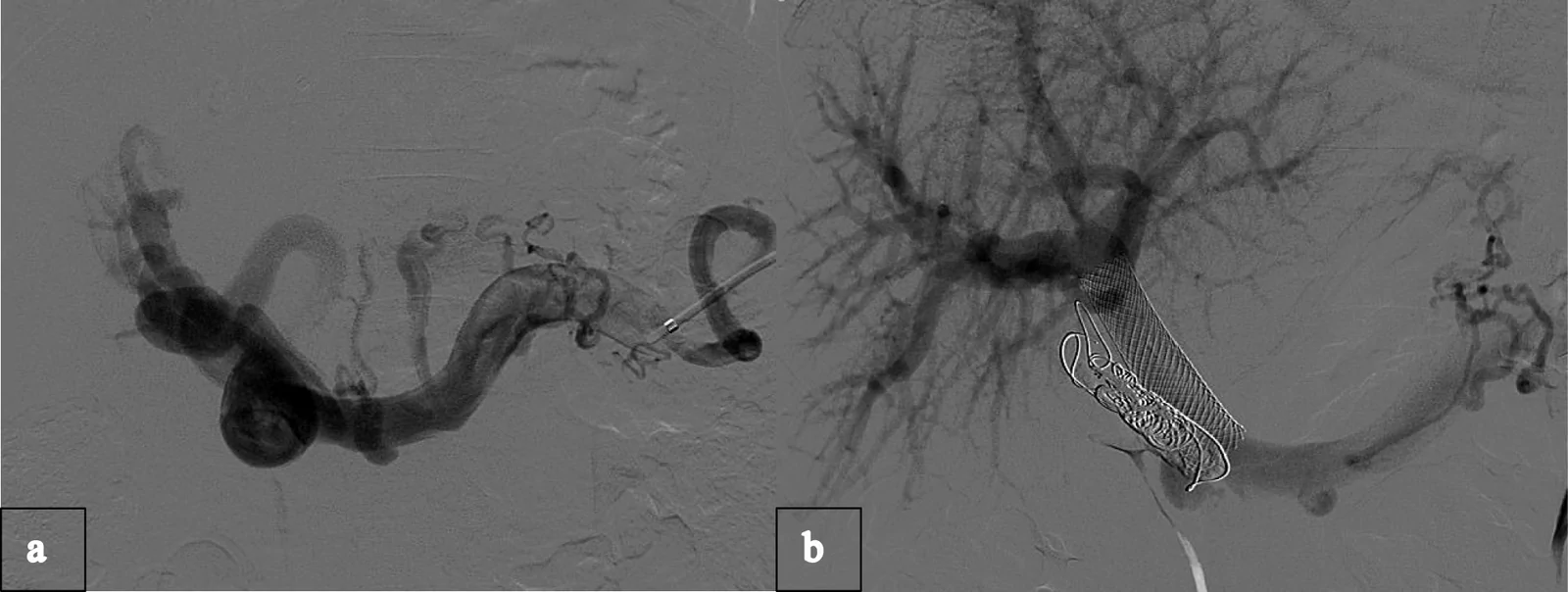
Transplenic portal vein recanalization with angioplasty, stenting and embolization of cavernoma. A 14-year-old boy with portal hypertension resulting from perinatal umbilical vein catheterization-related extrahepatic portal vein obstruction. **a** Anteroposterior transplenic digital subtraction venography image shows cavernous transformation of the portal vein with gastric varices. **b** Final anteroposterior digital subtraction venography image acquired after portal vein recanalization with angioplasty, stenting (Wallstent 12 × 40 mm, Boston Scientific) and embolization of the cavernoma

**Fig. 4:**
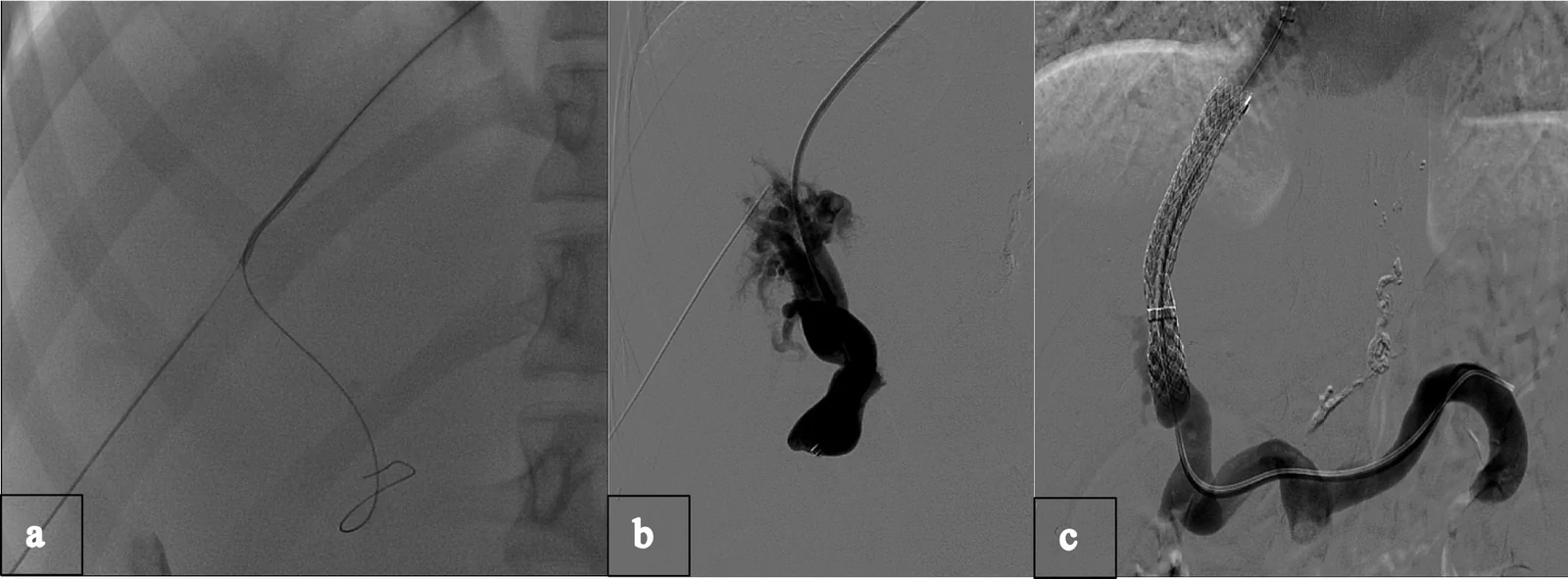
TIPS placement by combined transhepatic–transjugular approaches. A 13-year-old girl with portal hypertension resulting from extrahepatic portal vein obstruction who failed portal vein recanalization. **a** Anteroposterior fluoroscopic image shows the creation of a through-and-through transhepatic–transjugular access after ultrasound-guided targeting of the main cavernous vessel for TIPS landing. **b** Anteroposterior digital subtraction venography image acquired after navigation from the transjugular access into the cavernoma. **c** Anteroposterior digital subtraction venography image acquired after TIPS placement shows wide connection between the cavernoma and the splenic vein with effective decompression of the portal circulation

Portal hypertension in children can be cirrhotic or non-cirrhotic and the management varies accordingly. A rare non-cirrhotic cause is extrahepatic portal vein obstruction due to thrombosis, where interventional radiology (IR) could be a gamechanger, restoring the patency of the native portal system by portal vein recanalization. For all other cirrhotic and non-cirrhotic causes, growing evidence supports TIPS placement in children as safe and effective for emergent management of variceal bleeding, as well as for primary and secondary prevention of bleeding and other sequelae of portal hypertension [55, 56]. Technical challenges related to small patient size may be overcome by the use of intravascular ultrasound, transhepatic or trans-splenic “gun-sight” targeting (Fig. 2), and modifications to the transjugular cannula. Appropriate portal diversion may be achieved with nonstandard stent conduits where dedicated TIPS endografts are too large [57]. More recently, small series demonstrated the feasibility of TIPS in very small children weighing less than 10 kg, facilitated by percutaneous transhepatic establishment of the portosystemic connection [58, 59]. Altogether these advanced techniques may reduce the need to resort to surgical shunts which can be very challenging to create in small infants and children with elevated portal pressures, especially due to the risk of intraoperative bleeding. While several series on paediatric TIPS have been increasingly published, less is known about PVR. Only a few case reports and small series on feasibility have been published with limited data on outcomes and short follow-up [60, 61]. Compared to TIPS, PVR may completely restore a physiological condition, like the Meso-Rex bypass, provided that any other causes of portal hypertension, other than simple thrombosis, have been ruled out. Remarkably, either through a percutaneous transhepatic or transplenic access, PVR may restore physiological portal flow to the liver, resolving portal hypertension, even in those patients who are not eligible for, or have failed, the Meso-Rex bypass, due to obliteration of the Rex recessus (Fig. 3). If PVR fails, TIPS can still be created by using advanced percutaneous techniques (Fig. 4) as a rescue treatment to manage complications of portal hypertension. Limitations of the technique include exposure to significant amounts of ionizing radiation and iodinated contrast agents and the frequent need for additional procedures.

### Hepatic Lymphatic Embolization for Protein-Losing Enteropathy (PLE)

**Fig. 5:**
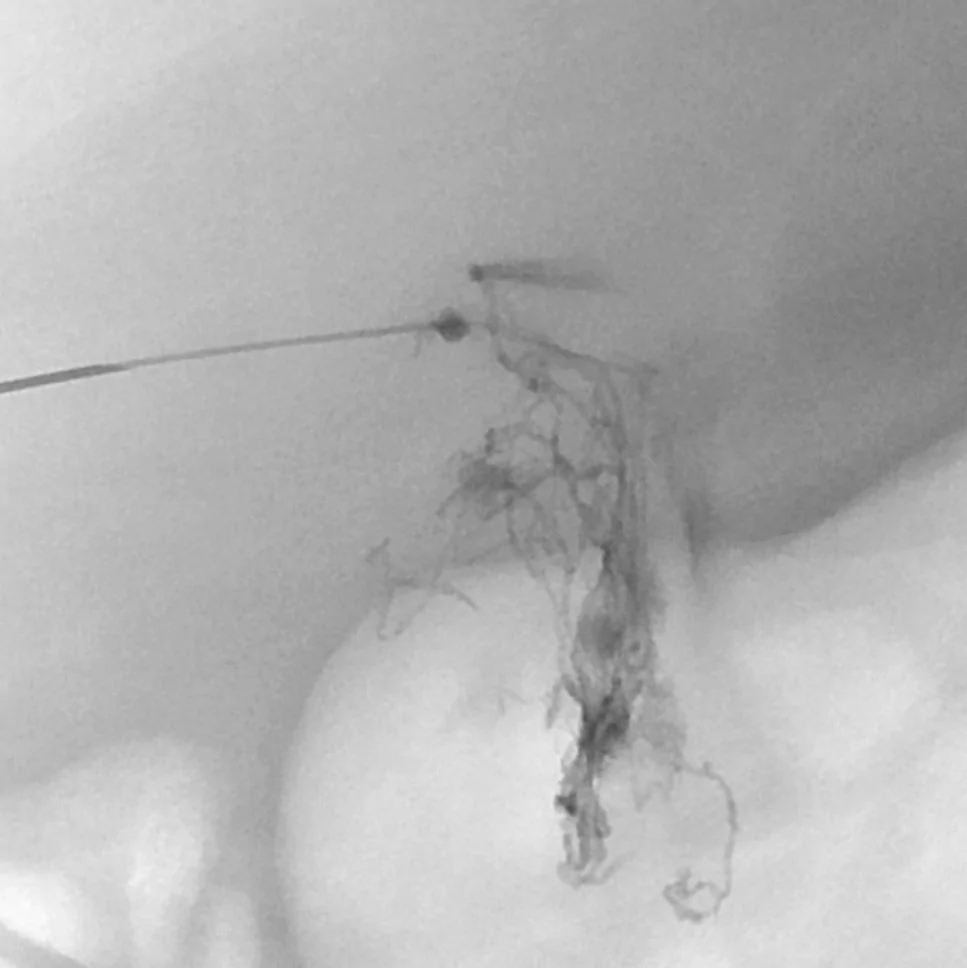
Transhepatic lymphatic embolization. A 12-year-old male with Fontan palliation and protein-losing enteropathy. Anteroposterior fluoroscopic image demonstrates percutaneous coaxial 21-g + 25-g needle access into the hilar hepatic lymphatics with cyanoacrylate embolization extending through deep/periportal lymphatic channels into dilated duodenal lymphatics

Fontan palliation of congenital heart disease induces chronically elevated right heart and, therefore, hepatic venous pressures, which in turn cause hepatic lymphatic engorgement and proximal small bowel intestinal lymphangiectasia. The resulting lymphorrhagia results in diarrhoea/steatorrhea and/or nausea and vomiting while the ongoing protein losses result in hypoproteinaemia, oedema, malnutrition, and immunosuppression [62]. Lymphatic embolization aimed at interrupting excessive flow through the deep hepatic lymphatic network effectively alleviates symptoms and sequelae of protein-losing enteropathy [63, 64]. Following US-guided percutaneous fine-needle access of the echogenic periportal tissue, lymphatic cannulation is confirmed with contrast injection. After ensuring the absence of nontarget vascular egress, embolization is performed with ethiodized oil and cyanoacrylate (Fig. 5).

### Percutaneous Transhepatic Cholecysto-Cholangiography (PTCC) for Biliary Atresia

Biliary atresia (BA) affects varying lengths of both intrahepatic and extrahepatic bile ducts, leading to cholestasis and persistent hyperbilirubinemia. If not promptly treated, BA leads to cirrhosis and liver failure. Historically, intraoperative cholangiography served as the gold standard in the evaluation of cholestatic jaundice. While confirmed cases of biliary atresia may proceed immediately to Kasai portoenterostomy, an operative approach may be considered an invasive and expensive method to exclude biliary atresia. In patients who present late (greater than 2 months of age), Kasai portoenterostomy may be futile but confirmation is still required for diagnosis and treatment planning. For these reasons, percutaneous transhepatic cholecystocholangiography (PTCC) was developed as a less invasive option and has since proven to be a safe and successful technique to exclude biliary atresia when noninvasive imaging and the clinical picture are inconclusive [65–67]. For patients with cholestasis due to inspissated bile rather than biliary atresia, the procedure may also be therapeutic [68].

**Fig. 6:**
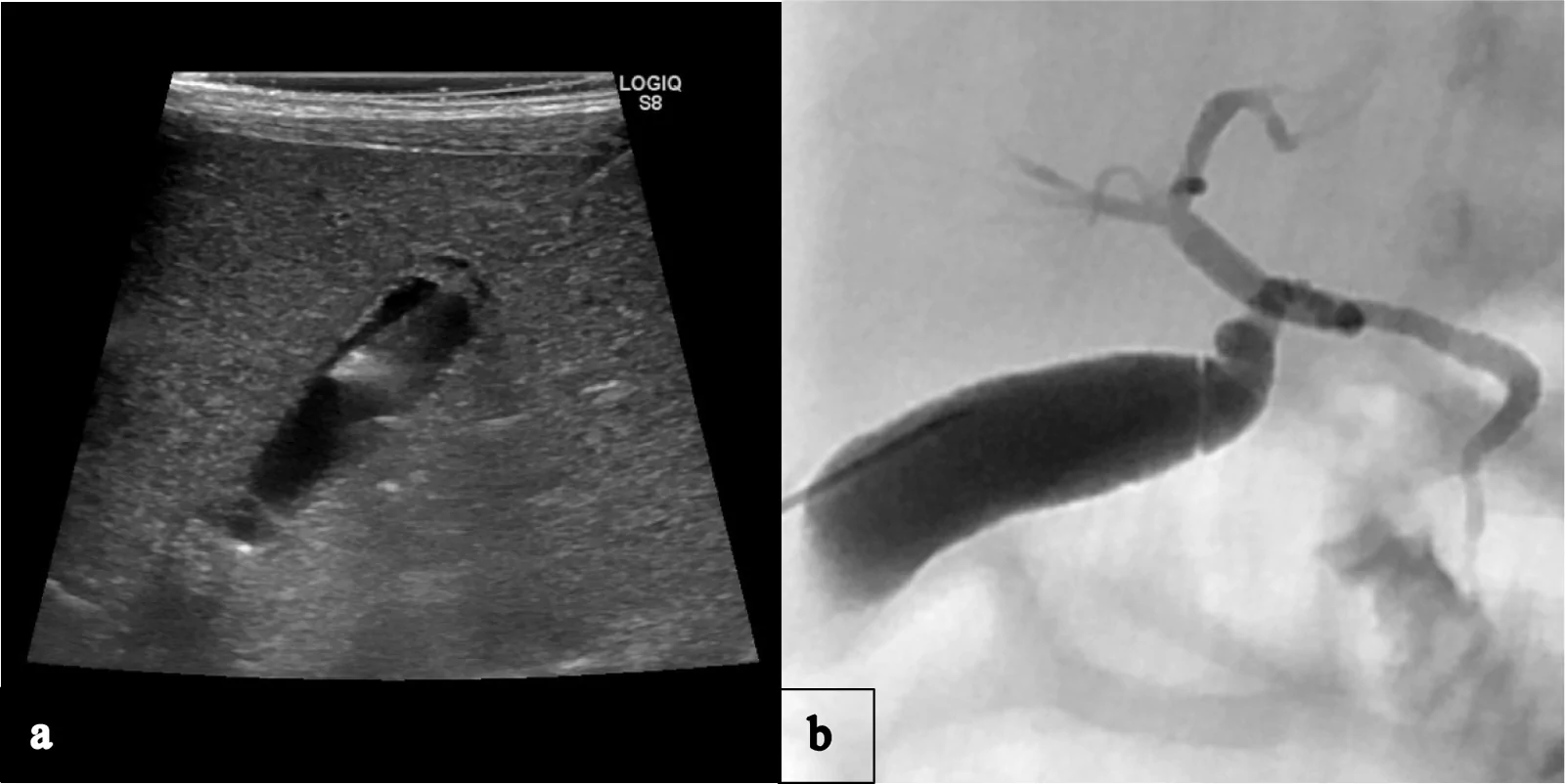
Percutaneous transhepatic cholecystocholangiography in a 2-week-old female with direct hyperbilirubinemia, acholic stools and hepatobiliary scan showing accumulation but no excretion of radiotracer. **a** Ultrasound image demonstrates a decompressed gallbladder accessed with a 25-gauge needle and distended slightly. **b** Fluoroscopic anterioposterior cholecystocholangiogram demonstrates continuity of the intra- and extrahepatic bile ducts with egress of contrast to bowel, excluding biliary atresia

**Fig. 7:**
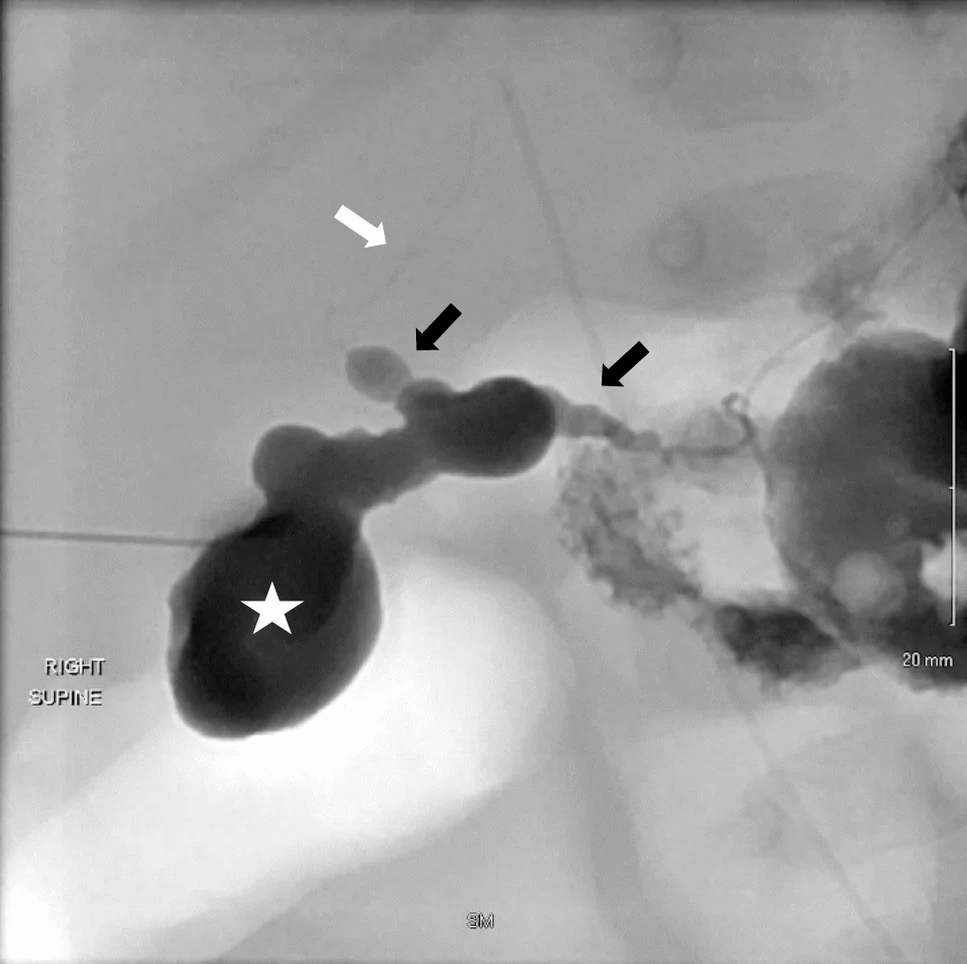
Cystic biliary atresia. 1-month-old infant with persistent hyperbilirubinemia and acholic stools. Anteroposterior cholangiogram following percutaneous 25-g needle access of the gallbladder demonstrates irregular contour of the gallbladder (white star) and cystic ectasia of the extrahepatic biliary tree (black arrows). Faint linear opacification within the liver (white arrows) could represent either lymphatic channels or markedly diminutive bile ducts

Infants with persistent hyperbilirubinemia are initially evaluated with ultrasound. Although no consensus exists, inconclusive ultrasound findings regarding biliary atresia may be assessed by Tc99m-Mebrofenin hepatobiliary scintigraphy (HIDA scan). For patients with a definitive or equivocal gallbladder on ultrasound and persistent retention of radiotracer on HIDA scan, PTCC is offered and may be performed in conjunction with ultrasound-guided needle biopsy of the liver for a comprehensive evaluation, thus avoiding laparotomy when a normal biliary tree is identified. Procedures are performed under general anaesthesia with prophylactic intravenous gram-negative antibiotic coverage. The gallbladder is accessed with a 25–27-gauge needle through the liver parenchyma. Inserting the needle along the long axis of the gallbladder may reduce the risk of back wall puncture and image contamination from hilar extravasation, while needle insertion from a transhepatic rather than transperitoneal approach may provide tamponade with lower rates of leaking after de-access. Contrast is gently introduced with continuous imaging until confirmation of the continuous intrahepatic ducts, common hepatic duct, and common bile duct to the duodenum (Fig. 6) or until segmental hypoplasia or non-visualization occurs despite adequate system distension (Fig. 7). The typical finding is a lack of reflux into the intrahepatic bile ducts, with or without visualization of the extrahepatic bile duct; filling of a continuous extrahepatic biliary tree only does not exclude biliary atresia [69]. The gallbladder is then aspirated, or alternatively flushed through to the bowel with saline, and the needle is removed.

### Biliary Complications After Liver Transplantation and Hepatobiliary Stenting

Biliary complications are a significant cause of graft dysfunction, graft loss, patient morbidity, and mortality following paediatric liver transplantation. Their overall incidence varies between 15 and 40% [70, 71]. They depend on the type of graft, surgical techniques, anatomical factors (such as the number of ducts), and coincident vascular complications, such as hepatic artery thrombosis. These complications may be early, usually resulting from bile leakage or stricture, or late, often due to biliary strictures.

Biliary strictures can be either anastomotic (either duct–duct or bilioenteric) or intrahepatic, or a combination of both. While anastomotic strictures may often relate to surgical techniques, intrahepatic strictures result from ischaemic injury, hepatic artery thrombosis, ABO incompatibility, acute rejection, or other post-operative complications. Noninvasive evaluation of strictures can be carried out by first-level assessment with ultrasound or by cross-sectional imaging with magnetic resonance cholangiography. Percutaneous transhepatic cholangiography is the preferred diagnostic and therapeutic option for children in most centres [72].

**Fig. 8:**
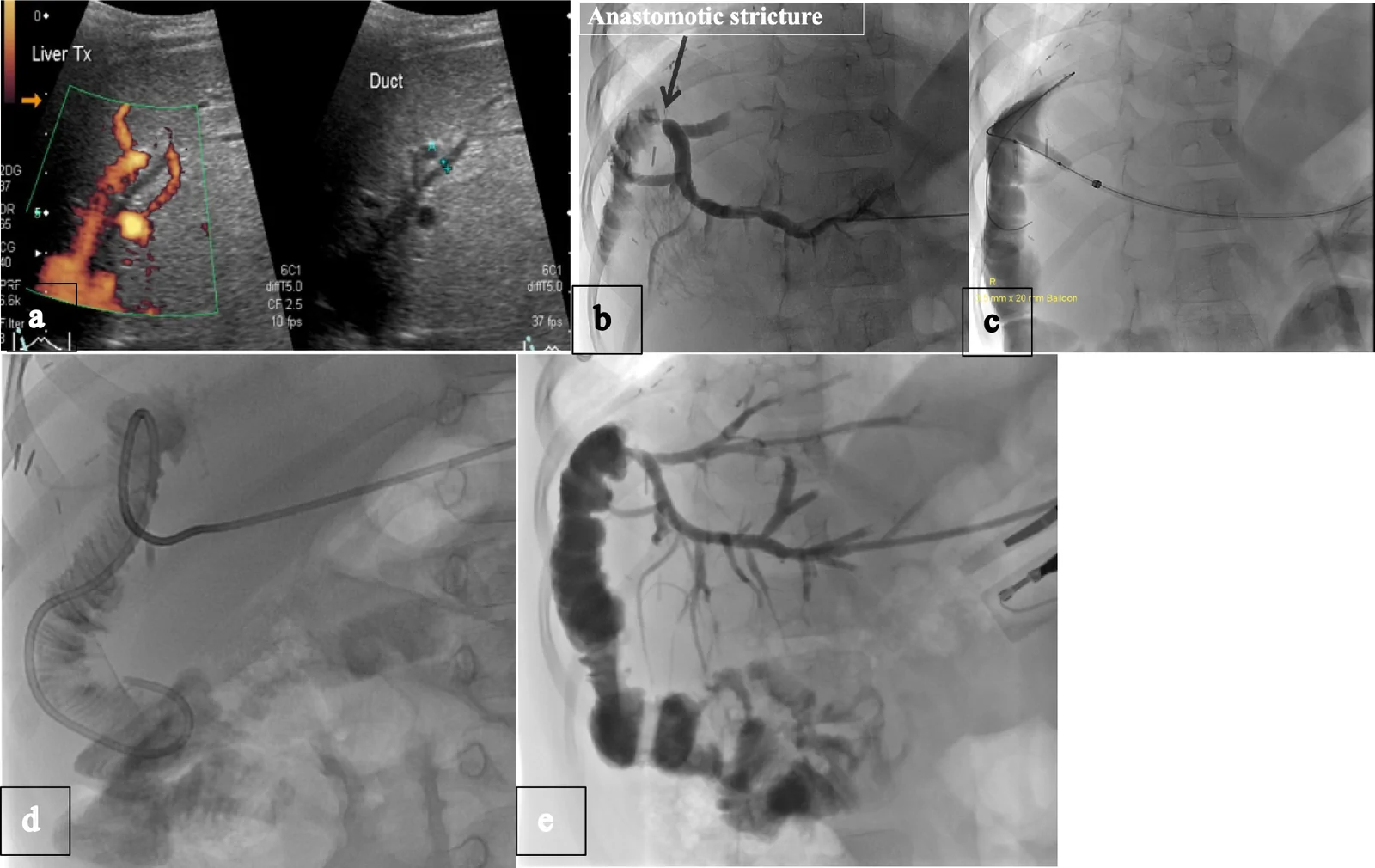
Percutaneous transhepatic cholangiography and balloon dilatation for anastomotic biliary stricture. A 6-month-old child, after living donor-related left lobe liver transplantation, presented with pruritus, rising bilirubin, and elevated gamma-glutamyl transferase. **a** Ultrasound of the liver shows dilated intrahepatic ducts. **b** and **c** Percutaneous transhepatic cholangiography reveals focal anastomotic stricture, which was crossed with a guidewire and treated with balloon dilatation. **d** Post-dilatation, an internal–external biliary drain was left across the anastomotic site. **e** A cholangiogram after 6 weeks of drainage shows wide patency of the anastomosis with resolution of duct dilatation

Biliary balloon dilatation (Fig. 8) has been extensively studied as a treatment for post-transplant biliary strictures, with reported technical success rates ranging from 75 to 90% and clinical success in 60–80% of cases [73]. However, high rates of stricture recurrence often necessitate multiple repeat procedures. Additionally, there is no consensus on the recommended duration of biliary drainage following biliary balloon dilatation nor on the type of biliary drainage (internal–external versus external) [74]. Potential complications of balloon dilatation include cholangitis, bleeding, and perforation, which can occur in up to 10–15% of cases.

**Fig. 9:**
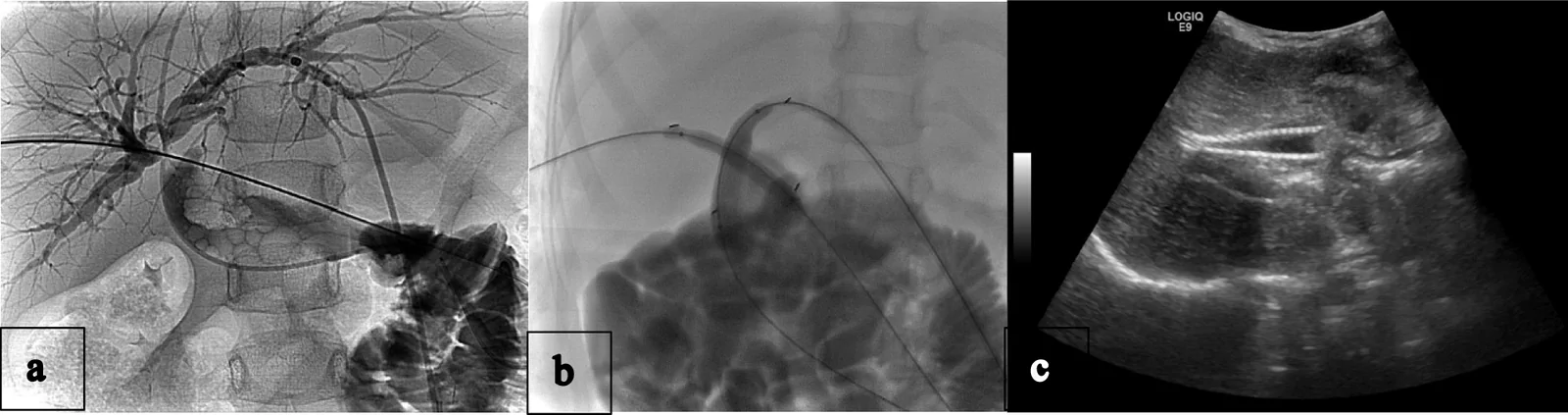
Percutaneous transhepatic cholangiography and biodegradable stents placement for bi-anastomotic hepaticojejunostomy stricture. A 3-year-old girl, after choledochal cyst resection and portoenterostomy, presented with jaundice. **a** Anteroposterior cholangiography image performed after bilateral transhepatic biliary catheterization shows dilated intrahepatic ducts with stricture involving the main ducts confluence. **b** Anteroposterior fluoroscopic cholangiography acquired during intrastent bilioplasty performed with the kissing-balloon technique after the deployment of two self-expandable polydioxanone biodegradable stents (ELLA-BD THP; Ella-CS Ltd.). The stents were applied on a custom-made basis since they did not yet have CE-mark approval, which has only recently been granted. **c** Ultrasound imaging demonstrates the stent's visibility throughout the degradation period, with structural integrity preserved for up to 8 weeks and a degradation profile spanning 3–6 months

While balloon dilatation has been the mainstay of treatment, emerging interventional radiology techniques have garnered attention for their potential to address the challenges associated with this approach. Newer approaches, such as biodegradable biliary stents (Fig. 9), have emerged as promising alternatives for the long-term management of biliary strictures in this patient population [75, 76]. A multicentre study which evaluated the safety and efficacy of biodegradable polydioxanone stents for treating benign biliary strictures in 102 paediatric liver transplant patients across five European centres showed a 76% success rate in preventing stricture recurrence, with minimal complications [77].

With the advent of interventional radiology-operated endoscopy, evaluation of strictures is feasible through direct visualization during cholangioplasty. Cholangioscopy-guided percutaneous biliary drainage can also be performed in liver transplant recipients who develop biliary cast syndrome in the peripheral ducts [78, 79]. Moreover, the use of cholangioscope-assisted laser incision (CALI) is described for the treatment of severe benign hepaticojejunostomy stenoses, avoiding surgical-related morbidity [80]. A pioneering study in children reports 100% technical and procedural success using percutaneous cholangioscopy for diagnosing and managing biliary pathology in four paediatric patients under age 8, across six procedures [81].

### Arterial Complications After Liver Transplantation

Despite advances in surgical techniques and medical management of post-transplant status, hepatic artery complications remain a significant problem affecting graft survival as well as post-operative morbidity, especially in children, in whom the reported incidence ranges between 1.7 and 16.3% [82].

**Fig. 10:**
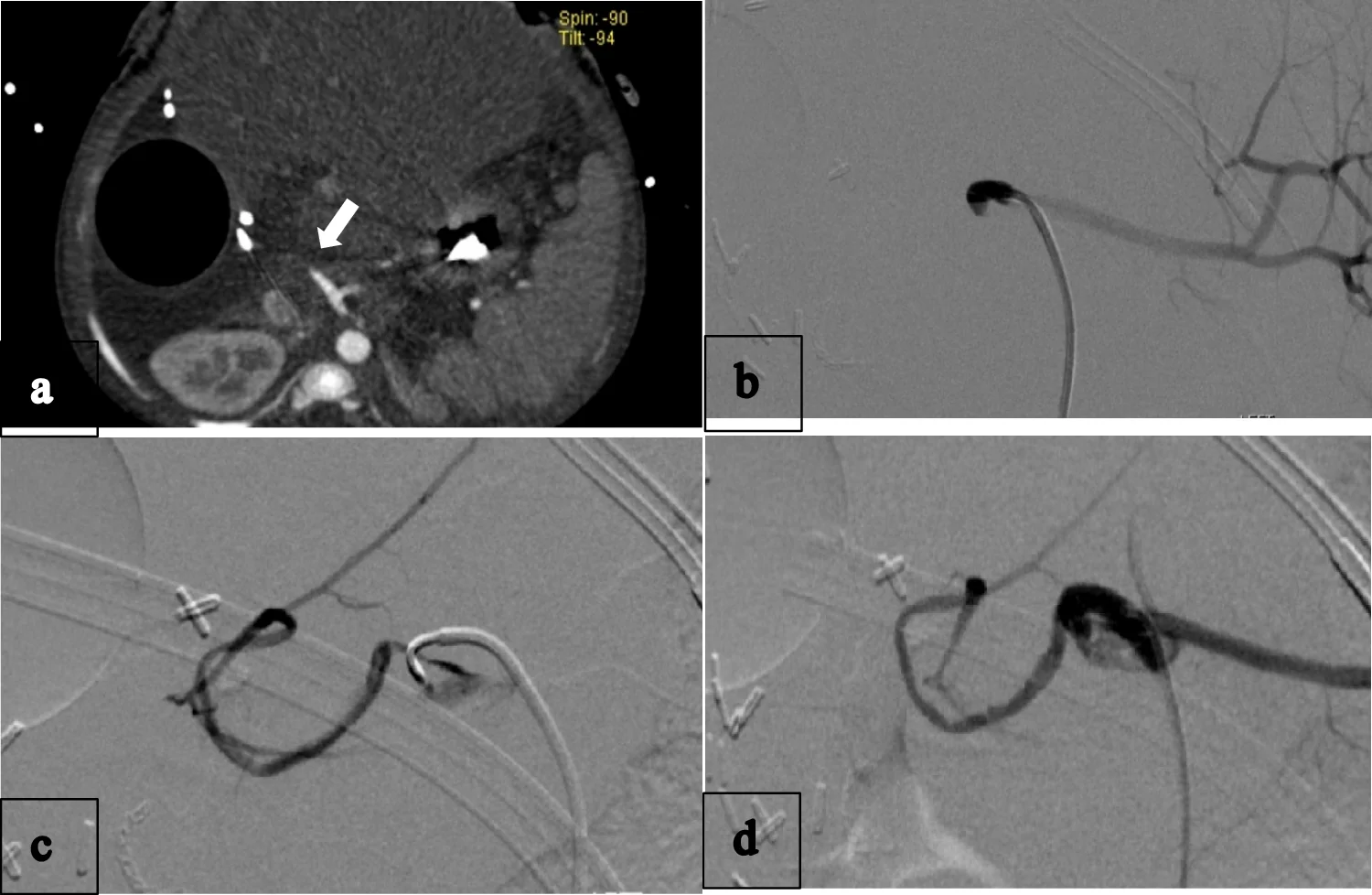
An 8-month-old child day-3 post-cadaveric split liver transplantation for biliary cirrhosis due to biliary atresia. **a** CT Angiogram of abdomen shows occluded (arrow) hepatic artery (HA) with non opacification of intrahepatic arterial tree. **b** Catheter angiography with selective coeliac injection confirms occlusion of HA. **c** Microcatheter selection of the origin of HA followed by intraarterial injection of tissue plpasminogean activator (tPA) thrombolysis. **d** Post-tPA coeliac angiogram shows restored patency of the HA

Hepatic artery thrombosis is among the most serious arterial complications, leading to biliary ischaemic strictures and graft loss, with reported incidences of up to 26% [80]. It often occurs as an early complication and may relate to risk factors such as prolonged cold ischaemic time, small hepatic artery (especially in split livers), ABO incompatibility, and acute rejection [81]. Its detection requires a high index of suspicion. Emergent revascularization is frequently indicated due to the high risk of biliary ischaemia/necrosis and the long-term complications associated with the development of biliary strictures. Protocol Doppler sonography allows early detection, which can then be confirmed via CT or arteriography. Treatment options include conservative management (anticoagulation), surgical thrombectomy and re-anastomosis, endovascular revascularization, or retransplantation in cases of graft loss [79]. Endovascular options have high success rates and include catheter-directed intraarterial thrombolysis (Fig. 10), either alone or in conjunction with angioplasty and/or stent placement; mechanical thrombectomy has also been employed in both adult and paediatric patients [82–84].

Hepatic artery stenosis occurs in about 5% of cases and is often a late complication [82]. The clinical presentation may be innocuous. It is suspected when the intrahepatic Doppler waveform displays a prolonged systolic acceleration time (≥ 0.08 s) and a low resistive index (RI) (< 0.5) (‘tardus et parvus waveforms’) associated with an increased peak systolic velocity (> 200 cm/s) at the anastomotic site [83]. Conservative management may be considered for mild, asymptomatic, or late-onset hepatic artery stenosis. Angioplasty, with or without stenting, is the preferred approach for early-onset cases associated with impaired liver function or biliary strictures. Endovascular and surgical revascularization demonstrate similar high technical success rates and favourable outcomes [84].

Hepatic artery pseudoaneurysm is a rare complication that may result from arterial injury, infection, or as a complication of angioplasty. Treatment options include coil embolisation, stent graft placement, or surgical resection, depending on the pseudoaneurysm's size and location [83, 85, 86].

Despite findings from multiple single-centre studies, the optimal management strategy for hepatic arterial complications and the effectiveness of various modalities remains uncertain [84, 87].

The HEPatic Artery stenosis and Thrombosis after liver transplantation In Children (HEPATIC) Registry has been designed to determine the overall incidence and the effectiveness of all treatment strategies for hepatic artery complications after paediatric liver transplantation by evaluating current management practices, including anticoagulant therapy, screening protocols, and assessment criteria, across multiple centres [82].

### Portal Vein Complications After Liver Transplantation

**Fig. 11:**
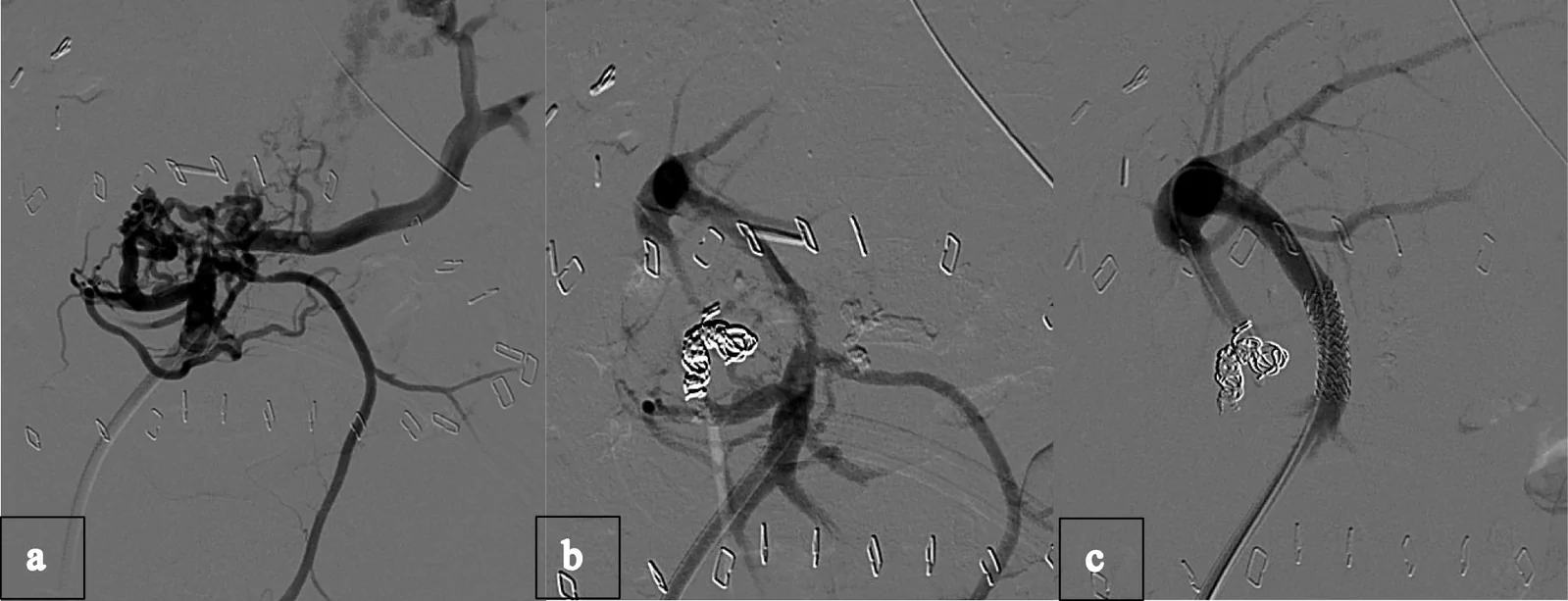
Transmesenteric recanalization of the portal vein anastomosis after liver transplantation. A 1-year-old boy with early acute portal vein obstruction after liver transplantation for hepatoblastoma. **a** Anteroposterior digital subtraction venography image acquired through the surgically inserted superior mesenteric vein sheath shows irregular obstruction at the level of the spleno-mesenteric confluence and amputation of the main portal vein, with opacification of developing cavernoma and gastric varices. **b** Anteroposterior digital subtraction venography image shows recanalization of the portal vein after angioplasty and embolization of the cavernoma (to prevent flow steal phenomenon); note that the main portal vein is still abnormally narrow. **c** Final anteroposterior digital subtraction venography image shows improved opacification of the intrahepatic portal vein and normal size of the extrahepatic after stenting (Herculink 8 × 18 mm, Abbott)

Portal vein complications after liver transplantation are uncommon but present significant morbidity as they usually manifest with life-threatening complications of portal hypertension, like variceal bleeding [88]. On routine post-transplant follow-up, the diagnosis of portal vein stenosis may be challenging due to the lack of universally accepted parameters for the definition of haemodynamically significant obstructions [89]; conversely, the detection of portal vein thrombosis is unequivocal. For both conditions, IR may offer resolutive treatments in the first instance, although resorting to IR is still not widely adopted, considering the high level of expertise required. Technical skills and familiarity with transhepatic or transplenic accesses to the portal vein are necessary. Surgical or percutaneous access to the mesenteric vein may also be considered if the other routes are not suitable (Fig. 11) [90]. However, transhepatic and transplenic puncture [91] proved to be safe for percutaneous catheterization of the portal system aimed at portal vein angioplasty or recanalization. One argument in the debate over treating portal vein stenosis after liver transplantation in children is whether to perform primary stenting or not. Bukova et al. [92] proposed primary stenting favouring single interventions with long-term patency results, while the largest body of literature provides evidence that angioplasty alone may be resolutive in up to 80% of cases [88], with additional procedures required for stenosis recurrence in a small proportion of patients. Unlike in the arteries, to achieve durable results with angioplasty alone in the veins, balloon catheters must be oversized to the target vessel. More data will soon be available from the PORTAL registry [93], which will provide new evidence about factors predisposing to portal vein obstruction and recurrence after angioplasty.

### Hepatic Veins Complications After Liver Transplantation

Hepatic venous complications following paediatric liver transplantation commonly manifests as outflow obstruction. Unlike other complications after paediatric liver transplantations, hepatic venous outflow obstruction (HVOO) is the less prevalent complication. However, it can result in graft dysfunction and possible loss due to liver congestion, portal hypertension, and cirrhosis. Its reported incidence ranges from 3.3 to 11%, being higher with living donor grafts and split cadaveric grafts compared to whole grafts in children [94, 95].

Surgical technical parameters can influence venous outflow obstruction, primarily the type of graft, the weight ratio of graft to patient, the number of hepatic veins and the type of anastomosis [96]. The common causes of acute or early hepatic venous obstruction often relate to anastomosis, which may involve a tight suture line with or without thrombosis. Other causes include torsion due to improper graft positioning, kinking of the hepatic vein or an interposed vein graft, stretching and twisting of the vein and the anastomotic segment caused by displaced liver grafts, discrepancies at the anastomotic level, graft oedema leading to swelling and extrinsic compression, graft regeneration, and intimal hyperplasia or fibrosis around the anastomotic sites [94].

Clinical presentation can be variable and may feature new-onset ascites, abnormal liver function tests, lower extremity oedema, portal hypertension, or renal insufficiency. The clinical severity of the venous outflow obstruction may not correlate well with the severity of the obstruction, requiring a high index of suspicion for diagnosis.

Ultrasound may be useful when suspecting early or acute HVOO and for long-term monitoring. Sonographic criteria indicative of HVOO include the persistent loss of normal phasicity, sluggish flow, absence of flow or reversed flow in the outflow veins, accelerated flow at the outflow anastomosis, and direct visualization of occlusion or stenosis on greyscale images [83, 97, 98].

Venography with measurement of the pressure gradient across the stenosis remains the standard for assessing HVOO. Quantifying the pressure gradient across the stenosis may help verify the lesion's significance. Though there is debate on the measurement's reliability and the exact value of the pressure gradient at which it would be considered abnormal, a gradient > 3 mmHg is generally regarded as significant [96, 99].

In paediatric patients with HVOO, balloon venoplasty is considered the first-line treatment option [95, 100]. Many may require multiple procedures, but most respond well to two or three dilatations [96, 101]. Excellent outcomes from treatment with balloon dilatation have been reported with long-term primary patency of over 50% and assisted primary patencies of 100% [99].

Stent placement has been used for recurrent stenoses with reproducible good results [102]. In adults, primary stenting has been successfully used with good patency rates. Ko et al. reported a 72% 5-year patency rate in their cohort of early HVOO, which included 3 children [94]. Although many studies have reported good results with low complications for stent placement, there is no consensus about the indications, type, size or timing of stent placement for HVOO. There is also no clear guideline regarding long-term anticoagulation for preventing thrombosis and in-stent stenosis in children who receive stent treatment [102]. Finally, when a liver transplant patient has HVOO, the possibility of not only anastomotic stricture but also VOD/SOS due to rejection should also be considered, which needs pathological diagnosis by liver biopsy [103].

### Lymphatic Complications After Liver Trasplantation

**Fig. 12:**
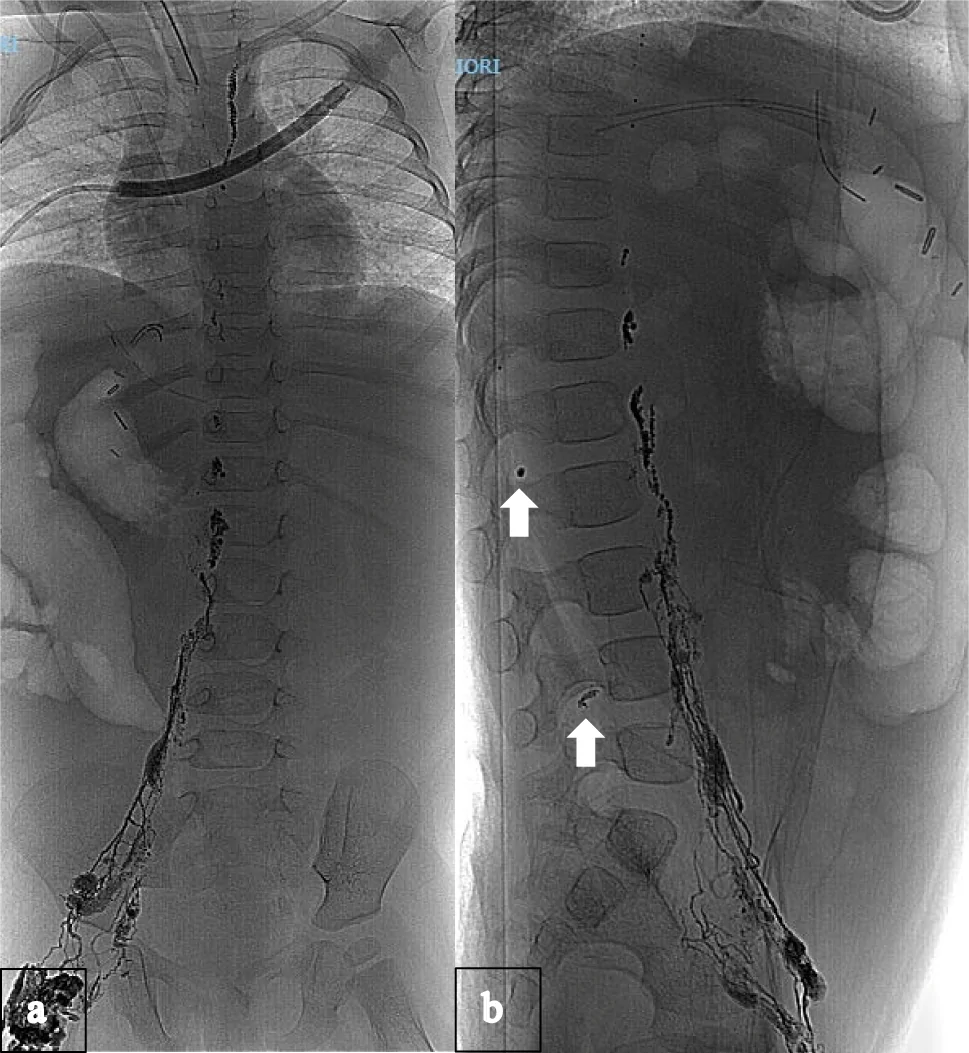
Intranodal lymphangiography. A 1-year-old girl suffering from refractory chylous ascites after split liver transplantation for biliary atresia. **a** Anteroposterior digital radiogram shows progression of ethiodized oil injected in a right groin lymph node up to the subclavian region. **b** Lateral projection radiogram shows extravasation of ethiodized oil droplets, consistent with lymphatic leakage. No embolization was attempted, as the lymphatic ducts were deemed not suitable for catheterization. Chylous ascites completely disappeared 1 week after the procedure

Chylous ascites is an uncommon complication following liver transplantation but its management is challenging as dietary restriction may significantly impair surgical recovery. As reported for the management of abdominal post-surgical biliary leakages resulting from different causes, lymphatic embolization may be considered a first therapeutic option, also in children [104–107] (Fig. 12).

### Emerging Technologies, Research Perspectives and Conclusions

Although the availability of devices specifically designed for paediatric use remains limited, technological innovation has led to significant improvements in endovascular equipment, which now features a sufficiently small profile suitable for small vessels. If necessary, even if used off-label, devices originally intended for cardiology or neuroradiology can be employed in paediatric peripheral applications. Furthermore, advances in ablation techniques for oncological diseases now offer instruments capable of treating tumourous lesions with a high safety profile, as in the case of cryoablation. Innovations in biliary interventions, such as biodegradable stents and novel cholangioscopy-assisted techniques, continue to improve outcomes in cases of complex post-transplant anatomy. However, greater commitment from industry is needed to develop instruments and kits specifically designed for use in low-weight patients (such as reduced-calibre kits and stents for creating TIPS in infants).

There is evolving data to support the role of transarterial therapies for liver tumours though the level of evidence is still limited by the retrospective nature of case series with lack of control groups. The same applies to other conditions, which are altogether rare in paediatric age. This highlights the need to establish multicentre and interdisciplinary research groups, fostering synergy between a multimodal diagnostic approach (as in the complex field of vascular anomalies) and treatments that integrate medical, interventional, and surgical therapies. IR is an essential component of multidisciplinary care, particularly for complex hepatic vascular anomalies, portal hypertension, and post-transplant complications. As technological advancements and growing clinical experience emerge, the scope and safety of these minimally invasive, organ-preserving interventions continue to enhance survival and long-term outcomes for both native and transplant liver disease in children.

## Declarations

### Human and Animal Participants

This article does not contain any studies with human participants performed by any of the authors. For this type of study, Institutional Review Board (IRB) approval was not requested.
